# Isolation, Cloning and Structural Characterisation of Boophilin, a Multifunctional Kunitz-Type Proteinase Inhibitor from the Cattle Tick

**DOI:** 10.1371/journal.pone.0001624

**Published:** 2008-02-20

**Authors:** Sandra Macedo-Ribeiro, Carla Almeida, Bárbara M. Calisto, Thomas Friedrich, Reinhard Mentele, Jörg Stürzebecher, Pablo Fuentes-Prior, Pedro José Barbosa Pereira

**Affiliations:** 1 Centro de Neurociências e Biologia Celular (CNC), Coimbra, Portugal; 2 Instituto de Biologia Molecular e Celular (IBMC), Universidade do Porto, Porto, Portugal; 3 BASF AG, Ludwigshafen, Germany; 4 Ludwig-Maximilian-Universität München, München, Germany; 5 Zentrum für Vaskuläre Biologie und Medizin, Erfurt, Germany; 6 Cardiovascular Research Center, Consejo Superior de Investigaciones Científicas (CSIC)-Institut Català de Ciències Cardiovasculars (ICCC), Barcelona, Spain; University of Queensland, Australia

## Abstract

Inhibitors of coagulation factors from blood-feeding animals display a wide variety of structural motifs and inhibition mechanisms. We have isolated a novel inhibitor from the cattle tick *Boophilus microplus*, one of the most widespread parasites of farm animals. The inhibitor, which we have termed boophilin, has been cloned and overexpressed in *Escherichia coli*. Mature boophilin is composed of two canonical Kunitz-type domains, and inhibits not only the major procoagulant enzyme, thrombin, but in addition, and by contrast to all other previously characterised natural thrombin inhibitors, significantly interferes with the proteolytic activity of other serine proteinases such as trypsin and plasmin. The crystal structure of the bovine α-thrombin·boophilin complex, refined at 2.35 Å resolution reveals a non-canonical binding mode to the proteinase. The N-terminal region of the mature inhibitor, Q16-R17-N18, binds in a parallel manner across the active site of the proteinase, with the guanidinium group of R17 anchored in the S_1_ pocket, while the C-terminal Kunitz domain is negatively charged and docks into the basic exosite I of thrombin. This binding mode resembles the previously characterised thrombin inhibitor, ornithodorin which, unlike boophilin, is composed of two distorted Kunitz modules. Unexpectedly, both boophilin domains adopt markedly different orientations when compared to those of ornithodorin, in its complex with thrombin. The N-terminal boophilin domain rotates 9° and is displaced by 6 Å, while the C-terminal domain rotates almost 6° accompanied by a 3 Å displacement. The reactive-site loop of the N-terminal Kunitz domain of boophilin with its P_1_ residue, K31, is fully solvent exposed and could thus bind a second trypsin-like proteinase without sterical restraints. This finding explains the formation of a ternary thrombin·boophilin·trypsin complex, and suggests a mechanism for prothrombinase inhibition *in vivo*.

## Introduction

Kunitz-type domains are common structural and functional elements found in extracellular proteins [1], which are usually associated with inhibition of trypsin-like serine proteinases [2]. Kunitz domains appear not only in single-domain proteins such as the paradigmatic member of the family, bovine pancreatic trypsin inhibitor (BPTI) [3], but also in multiple tandem repeats (e.g., the three consecutive domains that build up the major inhibitor of the coagulation cascade, tissue factor pathway inhibitor/TFPI) [4].

The crystal structure of the trypsin·BPTI complex [5] and ensuing structural analysis of other proteinase·Kunitz inhibitor pairs [6]–[10] provided major insights into the mechanism of serine proteinase inhibition, as well as into our current understanding of peptide bond cleavage by serine proteinases. In these enzyme·inhibitor complexes, the Kunitz domain inserts a protruding, disulfide-stabilised loop-the reactive-site loop-into the active-site cleft of its cognate proteinase. Essential elements of this binding mode, nowadays termed canonical, are that (i) the reactive-site loop adopts a substrate-like conformation by forming a short β-strand that aligns anti-parallel to proteinase residues S214–G216, with (ii) its P_1_ K/R residue occupying the S_1_ specificity pocket of the enzyme [11] (The chymotrypsin(ogen)-based numbering system is used throughout this work, as well as the Schechter and Berger nomenclature, in which substrate/inhibitor residues are denoted P_n_, …, P_1_, P_1_′, …, P_m_′, from N- to C-terminal, where P_1_-P_1_′ is the scissile peptide bond. The corresponding proteinase subsites that accommodate these residues are accordingly termed S_n_, …, S_1_, S_1_′, …, S_m_′.).

Hematophagous animals interfere with several mechanisms that support homeostasis in their preys. In particular, these parasites strictly require inhibition of blood clot formation to allow successful feeding and digestion. Thrombin (E.C. 3.4.21.5), as the ultimate proteinase of the coagulation cascade [12], represents an attractive and perhaps obligatory target for these animals. The proteinase possesses two characteristic surface areas enriched in basic residues located far from the catalytic centre, termed anion-binding exosites. Exosites I and II allow high affinity interactions with various macromolecular substrates and receptors. For instance, exosite I mediates selective binding of fibrinogen and protease activated receptors (PARs), as well as the endothelial receptor, thrombomodulin, while exosite II recognises glycosaminoglycans such as heparin and the platelet receptor GP Ib-IX-V (for a recent review on the role of thrombin exosites, see ref. [13]). Previously solved crystal structures of thrombin in complex with unrelated inhibitors derived from insects (e.g., rhodniin [14] and triabin [15]) or leeches [16], [17] have shown that exosites are also critical for inhibitor binding.

In spite of the large number of serine proteinases targeted by Kunitz domains, they were long considered ineffective as thrombin inhibitors. In fact, BPTI inhibits thrombin with a *K*_i_ value of ∼100 µM, ten orders of magnitude worse than trypsin [18], [19]. The crystal structure of human α-thrombin revealed a major reason for this observation: the reactive-site loop of a canonically binding Kunitz inhibitor would severely clash with the narrow active-site cleft of the proteinase, in particular with the unique insertion Y60A–W60D [20]. Another factor that disfavours binding of Kunitz inhibitors to thrombin is the presence of a glutamate at position 192, a position commonly occupied by a Gln residue. Accordingly, both the deletion of the triplet P60B–W60D and the single E192→Q mutation notably enhance thrombin affinity for BPTI and TFPI [21], [22].

The crystal structure of the (E192Q)thrombin·BPTI complex revealed large displacements of the insertion 60-loop, of up to 8 Å for the Cα atom of W60D [23]. This finding raised the following speculation about the role of thrombomodulin: binding to exosite I of the proteinase could induce repositioning of active site residues and surrounding loops, to allow processing of a substrate that is not cleaved by free thrombin, the anticoagulant protein C. Although the crystal structure of the thrombin·thrombomodulin complex did not reveal noticeable differences in the active site region of bound thrombin, when compared to other reported structures [24], it still remains theoretically possible that stronger exosite-binders could allosterically elicit large distortions at the catalytic centre.

The only thrombin inhibitor from the Kunitz family structurally characterised to date, ornithodorin from the soft tick *Ornithodorus moubata*, is comprised of two highly distorted Kunitz modules, none of which would be able to bind a serine proteinase according to the canonical mechanism [25]. Instead, ornithodorin docks with its C-terminal domain into the basic exosite I of thrombin, and utilises its N-terminal residues to bind across the active-site cleft. This N-terminal peptide adopts a parallel conformation with regard to β-strand S214–G216; the resulting non-substrate-like binding mode is thus similar to that of leech-derived inhibitors hirudin [16] and haemadin [17]. A non-canonical binding mode was also described for tick anticoagulant peptide (TAP), an *O. moubata* factor Xa (FXa) inhibitor [26].

The cattle tick *Boophilus microplus* (Acari: Ixodidae; recently re-classified as *Rhipicephalus microplus*) is one of the most widely distributed parasites of farm animals, causing an important economic impact both due to blood and milk losses and leather damage, and because it is a vector of major protozoan pathogens [27]. Proteinase inhibitors have long been recognised in eggs and larvae of *B. microplus*, and target trypsin, chymotrypsin, elastase, and plasma kallikrein [28]–[33]. Interestingly, some inhibitors were shown to prolong the activated partial thromboplastin time (APTT) for bovine blood. At most partial sequence data has been reported for these inhibitors, and their potential effect on blood coagulation remains to be clarified. More recently, the antimicrobial single-domain inhibitor, ixodidin, was isolated from the hemocytes of *B. microplus* and shown to target both elastase and chymotrypsin [34], while genomic analysis revealed the presence of additional Kunitz-type inhibitors that are similar e.g. to TFPI [35], [36].

Here we report the isolation, preliminary functional characterisation, cDNA cloning, and heterologous expression of a novel thrombin inhibitor from *B. microplus*, which we have termed boophilin. In contrast to all previously discovered natural thrombin inhibitors, boophilin potently inhibits additional trypsin-like serine proteinases, including trypsin and plasmin. Besides, and in line with these functional observations, its amino acid sequence is more closely related to canonical Kunitz inhibitors such as BPTI than to ornithodorin. These findings, in turn, suggested that boophilin could employ the energy liberated upon occupancy of exosite I to “loosen” thrombin's 60-loop, thus allowing insertion of its reactive-site loop in a canonical manner, and provoked a structural investigation of its mechanism of thrombin inhibition. However, the crystal structure of the bovine thrombin·boophilin complex, refined at 2.35 Å resolution, reveals a non-canonical, ornithodorin-like binding mode; the reactive-site loop of the N-terminal domain remains thus freely accessible to a second trypsin-like proteinase. Surprisingly, all elements involved in thrombin–inhibitor interactions occupy different relative positions when compared to the corresponding regions in the thrombin·ornithodorin complex.

## Results

### Isolation of a thrombin inhibitor present in engorged *B. microplus* ticks

Approximately 150 g of engorged ticks were used for inhibitor purification. A single peak containing thrombin inhibitory activity was detected on ion-exchange chromatography, and further purified by affinity chromatography on a thrombin-Sepharose column. This two-step purification protocol yielded about 50 µg of an essentially pure protein, as judged by PAGE analysis, which we have termed boophilin. This inhibitor displays an apparent molecular mass (M_r_) of ∼23 kDa (Supplementary Figure S1), as estimated by SDS-PAGE under reducing conditions, which almost doubles the value determined by mass spectrometry (13.9 kDa; Supplementary Figure S1). The anomalous migration in SDS-polyacrylamide gels appears to be related to the acidic character of the protein (calculated pI 4.1; see below), which could interfere with SDS binding. We also note that the unreduced protein migrates faster than reduced boophilin, suggesting the presence of a disulfide-knotted domain(s).

**Figure 1:**
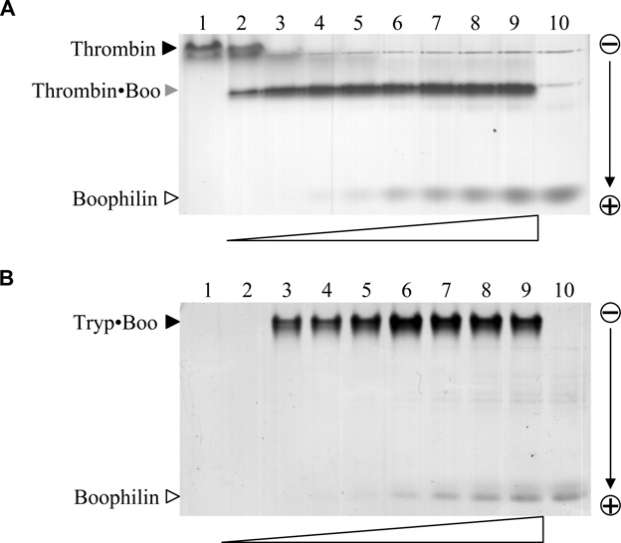
Boophilin forms stable stoichiometric complexes with free thrombin and trypsin. (A) Five-hundred nanograms of free human α-thrombin were incubated with increasing amounts of the purified inhibitor (from ≈140 ng, lane 2, to ≈2.1 µg, lane 9), and samples were resolved in a 10% polyacrylamide gel. Lanes 1 and 10 contain 500 ng thrombin and 2.1 µg boophilin, respectively. Notice formation of a single species corresponding to the 1:1 thrombin·boophilin complex (Thrombin·Boo). (B) Detection of equimolar trypsin·boophilin complex. Five-hundred nanograms bovine trypsin were mixed with increasing amounts of purified boophilin (from ≈100 ng, lane 2, to 1.2 µg, lane 9), and samples were separated in a 10% polyacrylamide gel. Lanes 1 and 10 contain 500 ng trypsin and 1.2 µg boophilin, respectively; cationic trypsin does not migrate into the gel. The stoichiometric trypsin·boophilin complex is marked (Tryp·Boo).

Boophilin was shown to double the thrombin clotting time of human plasma at an effective concentration of about 0.1 µM (mean of four determinations). Boophilin also showed a significant effect on the prothrombin time (effective concentration 0.4 µM; mean of four determinations), as well as a weaker effect on the activated partial thromboplastin time (effective concentration 1.7 µM, mean of two determinations). Assuming that the purified inhibitor binds bovine thrombin with tight-binding kinetics, we determined an apparent *K*_i_ of 1.8 nM. Further, we could show formation of a stable 1∶1 thrombin·boophilin complex in solution (Figure 1A). A free active site was required for complex formation, as no stable proteinase·inhibitor complex could be detected upon incubation with PPACK-inhibited thrombin (data not shown). These findings were confirmed by surface plasmon resonance experiments (Biacore); boophilin showed high affinity for free human α-thrombin, but bound only weakly to PPACK-thrombin or prethrombin-2 (not shown).

### Boophilin inhibits other serine proteinases in addition to thrombin

In striking contrast to other characterised thrombin inhibitors, boophilin could block the amidolytic activity of other trypsin-like serine proteinases, most notably trypsin and plasmin (Table 1). We examined trypsin inhibition in more detail and determined that boophilin inhibits this serine proteinase according to a slow-binding mechanism, with a second order rate constant k_2_/*K*_i_ of (3.08±0.60)×10^5^ M^−1^s^−1^ (mean of four determinations). In addition, we corroborated formation of a stable 1∶1 trypsin·boophilin complex in solution (Figure 1B). By contrast, the activity of other trypsin-like serine proteinases, and in particular of FXa, was only marginally affected (Table 1).

**Table 1:** Inhibition profile of boophilin

Enzyme	Inhibition of amidolytic activity (%) by		
	nBoophilin	rBoophilin	rBoophilin (C-term)
Trypsin	97	98	80
Factor Xa	10	0	0
Factor XIIa	n.d.	0	0
Plasma kallikrein	66	80	0
Factor VIIa	n.d.	57	0
Plasmin	87	95	25
u-PA	16	0	17
sc-tPA	n.d.	4	2
Tryptase	n.d.	8	8

Inhibitor samples were incubated with the nine serine proteinases listed, and the residual activity was measured after adding proteinase-specific chromogenic substrates, as follows: Pefachrome tPA (final substrate concentration 0.18 and 0.54 mM, respectively) to trypsin (final enzyme concentration 90 ng/ml) or sc-tPA (1.9 µg/ml); Chromozym X (0.36 mM) to FXa (0.11 U/ml); Pefachrome FXIIa (0.18 mM) to FXIIa (1.8 µg/ml); Chromozym PK (0.36 mM) to plasma kallikrein (0.52 µg/ml); Chromozym tPA (0.73 mM) to FVIIa (2.2 µg/ml); Chromozym PL (0.18 mM) to plasmin (91 µg/ml); Pefachrome uPA (0.18 mM) to u-PA (154 U/ml); and Chromozym TH (0.18 mM) to tryptase (0.11 µg/ml). Reactions were allowed to proceed for 2–6 min, and the effect of boophilin was estimated by setting the activity obtained without inhibitor as 100%. n.d., not determined. The concentrations used for boophilin samples were: native (n) inhibitor, 3.53 µM; recombinant (r) inhibitor (full-length), 2.36 µM; recombinant inhibitor (C-terminal domain), 15.2 µM.

### Boophilin is comprised of two Kunitz-type domains

Edman degradation of affinity-purified boophilin failed to reveal a signal for any amino acid residue, suggesting that the N-terminus of the inhibitor was blocked. However, sequences of internal boophilin regions could be obtained form chemically or enzymatically generated peptides. Comparison of these sequences against the Swiss-Prot database revealed strong similarities to Kunitz-type proteinase inhibitors from different species, in particular to the prototypic member of the family, BPTI (Supplementary Table S1; see also below). In light of the molecular mass determined with MALDI-MS (matrix-assisted laser desorption/ionisation mass spectrometry), this partial sequence data strongly suggested that boophilin comprised two Kunitz-type domains (M_r_≈6,500 Da) connected by a short linker.

To continue structural and functional characterisation of boophilin, we decided to clone and express the recombinant inhibitor. For this purpose, we constructed a cDNA library of unfed *B. microplus* containing 1.8×10^6^ independent clones, with inserts ranging from 0.5 to 4.0 kb in size. Degenerate oligonucleotides *Lys-C11Fwd* and *Lys-C15Rev* were designed (Supplementary Table S2), exploiting the presence of a triplet of aromatic residues between the second and third cysteine residue in each of the two boophilin Kunitz-type domains. These primers were used to amplify a single ∼300-bp cDNA fragment by PCR on randomly primed cDNA derived from poly(A)^+^ RNA of engorged ticks. The size of this PCR product is in good agreement with the figure expected for a DNA fragment coding for the sequenced boophilin fragment (see above). A band of the same size was observed when performing PCR reactions on the cDNA library phage stock, confirming boophilin expression in unfed animals as well.

**Figure 2:**
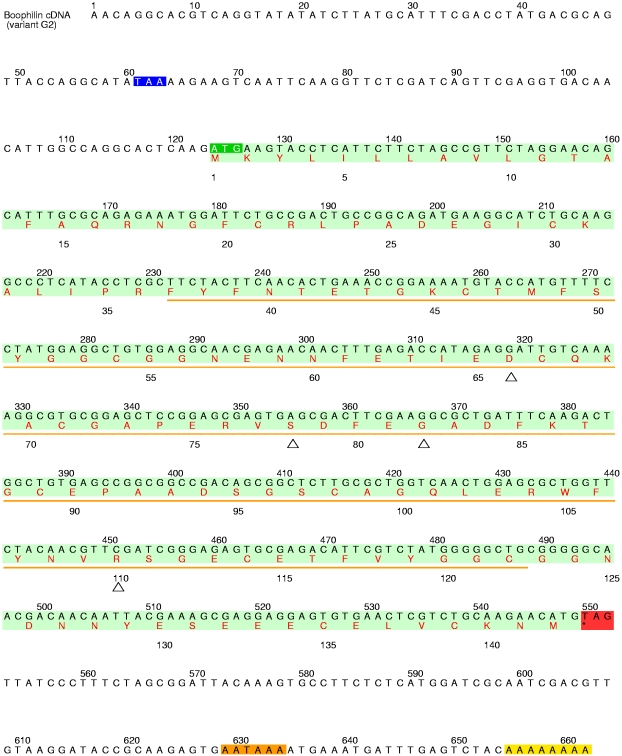
Complete nucleotide sequence of the cDNA encoding for boophilin variant G2 and predicted amino acid sequence. The coding region is shaded in light green, the start codon in dark green, the stop codon in red, the polyadenylation signal in orange, and the short poly(A) tail in yellow. The deduced amino acid sequence of pro-boophilin is shown in red below the nucleotide sequence, in one-letter code. An orange line indicates the fragment amplified by PCR. The in-frame stop codon that precedes the start codon for pro-boophilin is shaded blue. The deduced amino acid sequence of boophilin (142 residues) would correspond to a polypeptide with a higher molecular mass than that experimentally determined by MALDI-MS. However, if one considers the presence of a 15-residue pro-peptide, the resulting mature inhibitor would have a theoretical mass of 13,950 Da, in reasonable agreement with the experimentally determined value of 13,964 Da. (Notice that no putative *N*-glycosylation sites are present in the boophilin sequence). Further, the mature protein starts with a glutamine residue that could spontaneously cyclise to pyrrolidone carboxylic acid (5-oxo-proline). This modification would explain the blocked N-terminus observed in native boophilin, and has previously been reported in other Kunitz inhibitors such as an BPTI variant from bovine lung [74]. Open triangles indicate the positions where discrete differences exist in the amino acid sequences of the two boophilin variants.

Screening of the cDNA library with the labelled amplified fragment allowed identification of six independent positive clones, which were isolated and sequenced. These independent cloning events confirmed the presence of two boophilin variants (in the following termed G2 and H2), as suggested by the protein sequencing results. The complete nucleotide and deduced amino acid sequence of variant G2 is presented in Figure 2. The DNA sequences of the two cloned boophilin variants differ at twelve independent positions. All of these differences arise from point mutations and result in six amino acid exchanges in the encoded proteins: two at positions 3 and 4 of the propeptide, and four in the mature protein. However, these substitutions are conservative (D66→E in the N-terminal Kunitz domain, S78→N and G82→S in the linker region, and R110→Q in the C-terminal domain), and are thus not expected to interfere with the inhibitory potential of boophilin. Rather, they could reflect the adaptation to different preys or the response to immunological pressure, as observed in other blood-feeding animals.

**Figure 3:**
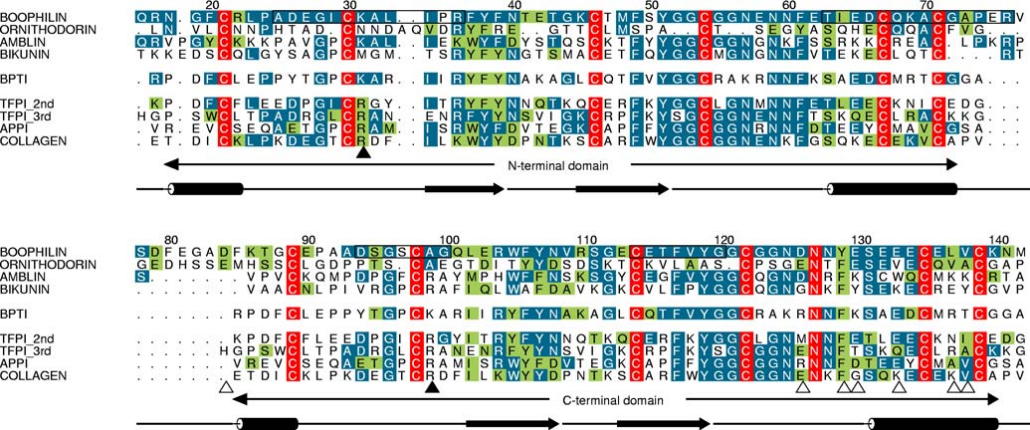
The two boophilin domains are more closely related to canonical Kunitz modules than to ornithodorin. Comparison of the amino acid sequence of mature boophilin with three other two-domain inhibitors, ornithodorin, amblin, and bikunin; the unique C-terminal extension in amblin has been omitted for clarity. Also included in the alignment are the sequences of BPTI and of the closest related human Kunitz modules structurally characterised so far (two copies, one aligned with each Kunitz module of boophilin): The second and third TFPI domains (1TFX [6] and 1IRH [75], respectively), the single Kunitz domain of APP (1AAP) [39], and the C-terminal Kunitz domain of type VI collagen (2KNT) [76]. Numbers refer to the full-length sequence of boophilin. Residues conserved throughout are white with red shading; residues identical to boophilin are white with blue shading; conservatively replaced residues are shaded green. The four major antigenic determinants in boophilin are boxed. The secondary structure elements of boophilin are shown below the alignment; β-strands are represented as arrows and α-helices as cylinders. The P_1_ residues within the reactive-site loops are indicated with black arrowheads; open arrowheads point to boophilin/ornithodorin residues involved in important contacts with thrombin exosite I. Notice that several exosite-interacting residues are conserved or conservatively replaced in boophilin, while they are mostly non-conservatively replaced in amblin (e.g., E130→K, L137→K).

The sequences of mature boophilin and of another related, recently reported thrombin inhibitor, amblin [37], are compared to the previously characterised ornithodorin and to BPTI in Figure 3. It is immediately apparent that both boophilin domains are much more closely related to BPTI and other canonical Kunitz modules than to ornithodorin. Twenty-six residues of the N-terminal and 22 residues of its C-terminal domain are identical to BPTI, whereas the corresponding domains in boophilin and ornithodorin share only 12 or 13 identical residues. Furthermore, cysteine residue spacing is exactly conserved in boophilin and BPTI. Most relevant for serine proteinase inhibition, the sequence of the reactive-site loop in the N-terminal domain (G28-I-C-K↓A-L-I-P35; K31 corresponds to the P_1_ residue) closely matches the corresponding G12-P-C-K↓A-R-I-I19 sequence in BPTI and other Kunitz inhibitors, and the secondary binding loop (F49–C54) is even better conserved. These sequence similarities between boophilin and BPTI contrast with the multiple insertions and deletions found in ornithodorin (Figure 3), which altogether hinder its binding to serine proteinases according to the canonical mechanism [25].

### Recombinant expression of boophilin and further functional characterisation

A cDNA fragment coding for the full-length mature protein (residues Q16 to M142, variant H2) was amplified by PCR and subcloned into the pRBI-DsbC expression vector, as a fusion construct with the OmpA periplasmic signal sequence (oligonucleotides used for subcloning are given in Supplementary Table S2). *Escherichia coli* DH5α cells were transformed with the expression construct and used for heterologous expression of recombinant boophilin, which was found to be correctly processed and active in the periplasmic space of the bacteria (not shown). The recombinant product was purified to homogeneity by anion-exchange chromatography on Q-Sepharose FF and ResourceQ columns, and authenticity was verified by N-terminal sequencing.

To confirm production of a correctly folded inhibitor we have verified the inhibitory effect of recombinant boophilin on blood coagulation (concentrations needed for doubling clotting times in the absence of the inhibitor: thrombin time, 0.045 µM; prothrombin time, 0.160 µM; APTT, 0.880 µM), as well as its ability to inhibit thrombin (*K_i_* = 3.4 nM). Next, we verified the inhibition of two other trypsin-like serine proteinases (trypsin and plasmin), as well as a partial effect on plasma kallikrein (80% inhibition at 2.36 µM, still 32% inhibition at 0.47 µM; compare Table 1). Finally, we have detected a weak inhibitory effect on FVIIa (57% inhibition at 2.36 µM). In contrast, other serine proteinases (FXa, FXIIa, uPA, sc-tPA, and tryptase) were not inhibited by boophilin. The second-order inhibition constants against trypsin and plasmin, determined using recombinant boophilin, are (3.84±0.73)×10^5^ M^−1^s^−1^ (mean of three determinations) and (2.59±0.22)×10^5^ M^−1^s^−1^ (mean of four determinations), respectively, and thus similar to the values reported for BPTI [18], [38]. All inhibitory properties of recombinant boophilin are similar to those observed for the inhibitor isolated from natural sources. Small differences observed are likely to be accounted for by the presence of a mixture of boophilin isoforms, in the native material.

By contrast, the isolated C-terminal boophilin domain, serendipitously obtained by cleavage of the A83–D84 peptide bond in the interdomain linker of recombinant boophilin by an endogenous proteinase of *E. coli*, showed essentially no effect on either thrombin time, prothrombin time or partial thromboplastin time at a concentration of 1 µM. Only at a rather high protein concentration of 20 µM, a marginal increase (30%) in thrombin time was observed. In addition, none of the serine proteinases tested was significantly inhibited by the isolated boophilin domain (Table 1). Clearly, presence of an alanine residue at the P_1_ position in the C-terminal boophilin domain (Figure 3) strongly disfavours its canonical binding to trypsin-like serine proteinases. Altogether, our results suggested that boophilin N-terminal domain inhibited a subset of trypsin-like serine proteinases according to the canonical mechanism, with residue K31 occupying the S_1_ pocket of the bound enzyme.

### Thrombin-bound boophilin retains the capability to interact with other serine proteinases

**Figure 4:**
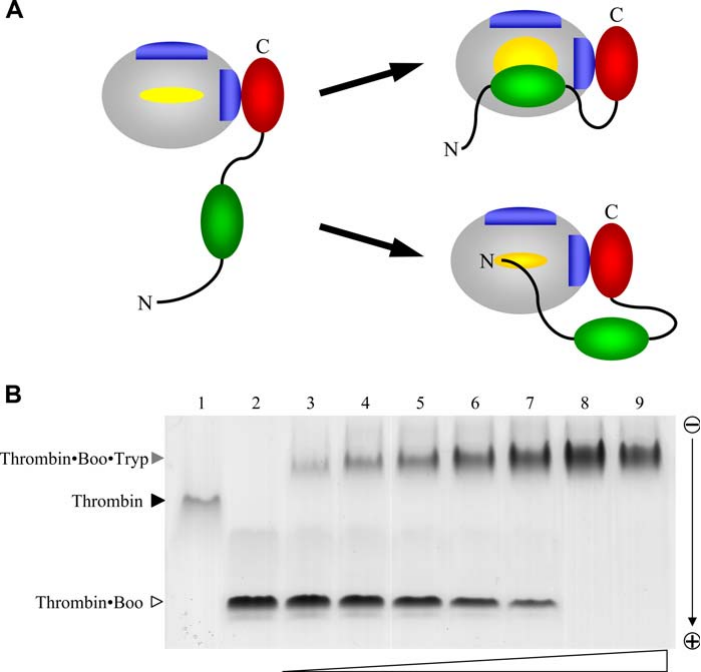
Thrombin-bound boophilin retains the ability to interact with other serine proteinases. (A) Schematic representation of two hypothetical thrombin·boophilin complexes. In the upper, BPTI-like mechanism, binding of the C-terminal boophilin domain to exosite I promotes extensive rearrangements of loops surrounding the active site to allow insertion of the N-terminal domain in a canonical manner. In the alternative, ornithodorin-like mechanism, exosite engagement is not associated with important modifications of the thrombin active site region, which is occupied by the N-terminal peptide of the inhibitor in a parallel manner. (B) Demonstration of thrombin·boophilin·trypsin ternary complex formation via native gel electrophoresis. One µg human α-thrombin·boophilin complex was incubated with increasing amounts of bovine trypsin (from ≈300 ng, lane 3, to ≈6 µg, lane 9), and samples were resolved in an 8% polyacrylamide gel. Lanes 1 and 2 contain 1 µg thrombin and 1 µg thrombin·boophilin complex, respectively; the newly formed species corresponds to the ternary complex.

The functional evidence presented above, combined with structural information for thrombin·ornithodorin [25] and thrombin(E192Q)·BPTI complexes [23], immediately suggested two possible modes for boophilin binding to cognate thrombin (Figure 4A). In both cases, the highly acidic boophilin C-terminal domain (11 aspartate/glutamate residues outbalance four basic residues) would interact with thrombin's positively charged exosite I. However, the two models differ with regard to the region of the N-terminal domain that blocks the active site of the proteinase. Binding in a canonical manner, i.e. via reactive-site loop requires major rearrangements of loops surrounding the active centre, and in particular of the 60-loop. By contrast, in the ornithodorin-like conformation only the N-terminal peptide of the inhibitor interacts with thrombin active site, which is essentially unmodified compared to the free enzyme. Preliminary modelling experiments indicated that both conformations could be plausibly adopted in the binary thrombin·boophilin complex, given the considerable length of the interdomain linker.

To discriminate between these opposite binding modes, we reasoned that the reactive-site loop of the bound inhibitor would only be exposed in the ornithodorin-like conformation to allow targeting of a second serine proteinase. This possibility was analysed using trypsin as a probe. Indeed, we could demonstrate formation of a ternary thrombin·boophilin·trypsin complex in solution (Figure 4B), in agreement with a non-canonical binding mode. We conclude that the N-terminal boophilin domain can simultaneously engage thrombin through its N-terminal peptide, and a second trypsin-like serine proteinase in a canonical manner.

### Thrombin structure is not altered in its complex with boophilin

**Figure 5:**
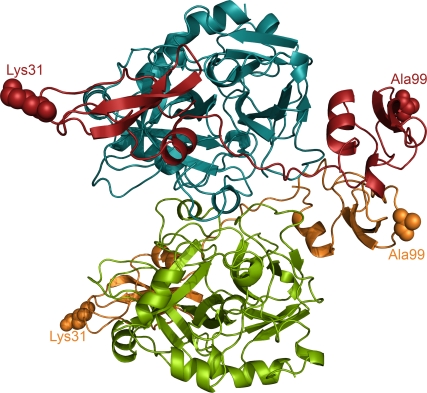
Three-dimensional structure of the α-thrombin·boophilin complex. The crystallographic dimer present in the asymmetric unit is shown as a ribbon plot; thrombin molecules are coloured blue and green and boophilin molecules are coloured red and orange. Notice that the reactive-site loops of both inhibitor domains point away from the proteinase moiety; the corresponding P_1_ residues, ^B^K31 and ^B^A99, are shown as space-filling models. Within the dimer interface, direct hydrogen bonds are formed between the side chain of ^B^S97 and the main-chain carbonyl of ^B^G124 located in opposite C-terminal domains of boophilin, and between the side chains of ^T^D21 and ^T^Q14A.

To verify the non-canonical mechanism of thrombin inhibition by boophilin, we decided to solve the three-dimensional structure of the equimolar bovine thrombin·boophilin complex. The complex crystallises as a dimer in the asymmetric unit (depicted in Figure 5). This non-crystallographic dimer is maintained by a small number of water-mediated polar interactions, mostly connecting opposite thrombin molecules. The small size of buried surface (∼400 Å^2^), as well as the character of the residues involved in dimer formation and the overall nature of their interactions are clearly compatible with a crystallisation-induced contact, but not with a physiologic dimer. Furthermore, boophilin behaves as a monomer in gel filtration chromatography (data not shown). The two complexes are virtually identical (r.m.s.d. of 0.58 Å for 419 equivalent Cα positions), thus only complex A (thrombin chains A and B and boophilin chain E) will be described.

By contrast to the major rearrangements of loops surrounding the active site centre observed in the (E192Q)thrombin·BPTI complex, the structure of thrombin is virtually unaltered upon boophilin binding. In comparison to the unliganded molecule (e.g., free α-thrombin from PDB entry 1MKX) 279 Cα atoms of the proteinase can be aligned with a r.m.s.d. of only 0.59 Å.

### The two boophilin domains possess a canonical Kunitz fold

**Figure 6:**
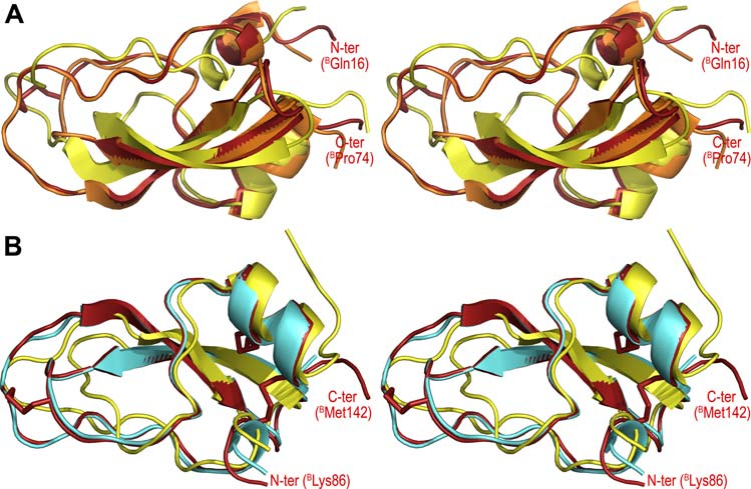
Boophilin is structurally more related to canonical Kunitz modules than to the distorted ornithodorin domains. Stereo plots showing the three-dimensional structure of (A) boophilin's N-terminal domain (red) overlaid on those of the second TFPI domain, as seen in its complex with porcine trypsin (orange, PDB entry 1TFX) [6], and of the N-terminal ornithodorin domain (yellow, 1TOC) [25], and (B) boophilin's C-terminal domain (red) superimposed on those of APP single Kunitz domain (cyan, 1TAW) [8], and the C-terminal ornithodorin domain (yellow, 1TOC) [25]. The N and C termini of boophilin are labelled and its disulfide bonds are shown as sticks. Notice that in spite of significant sequence similarity between the carboxy-terminal domain of boophilin and other Kunitz-type inhibitors (Figure 3), with strict conservation of cysteine spacing, its putative P_1_ residue is nevertheless an alanine (^B^A99), thus precluding inhibition of trypsin-like serine proteinases.

As implied form the sequence conservation (Figure 3), boophilin consists of two canonical Kunitz domains that are connected by a 10-residue linker. The N-terminal Kunitz module is most closely related to the second TFPI domain [6] (r.m.s.d. of 0.57 Å for 56 equivalent Cα atoms). In particular, it is noteworthy that the reactive site loops of both molecules overlap perfectly (Figure 6A). Ornithodorin, on the other hand, with its non-canonical cysteine spacing, deviates significantly from boophilin (r.m.s.d. of 1.75 Å for only 29 equivalent Cα atoms), with major differences clustering at the secondary structure elements and the reactive site loop.

From a structural point of view, the C-terminal domain of boophilin is closest to the Kunitz module found in amyloid precursor protein-APP [39] (r.m.s.d. of 0.48 Å for 53 equivalent Cα atoms; Figure 6B), but there is nevertheless a reasonable structural fit with the C-terminal domain of ornithodorin (r.m.s.d. of 1.40 Å for 44 equivalent Cα atoms), with conservation of all secondary structure elements. Once again, the largest deviations between both structures are in the vicinity of the reactive site loop, which in boophilin strictly follows the canonical path.

The solvent-exposed surface of boophilin is rather negatively charged, especially at the C-terminal domain, which is similar to that of ornithodorin (Supplementary Figure S2). Upon closer inspection, however, we noticed that the N-terminal domain of boophilin displays a more balanced distribution of opposite charges, in contrast to the net negative potential of the first ornithodorin module, both at the solvent-exposed and the proteinase-contacting surfaces (Supplementary Figure S2). As for the C-terminal Kunitz module, there is a comparable distribution of negative charges at the solvent-exposed surface for both inhibitors (Supplementary Figure S2).

### Significantly different arrangements of boophilin and ornithodorin in their complexes with thrombin

**Figure 7:**
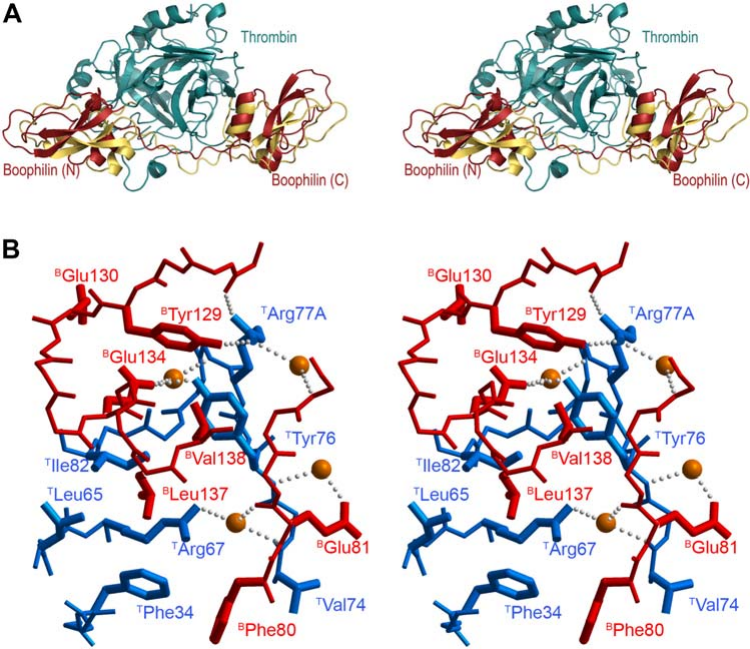
Different orientations of the Kunitz domains in thrombin complexes with boophilin and ornithodorin. (A) Stereo plot showing a comparison of thrombin·boophilin (blue/red) and thrombin·ornithodorin (blue/yellow) complexes, after overlaying both thrombin moieties. Notice the large displacements of the corresponding N- and C-terminal Kunitz modules relative to each other. (B) Stereo close-up highlighting the docking of the C-terminal domain of boophilin (red) to thrombin (blue) exosite I. Water molecules are represented as orange spheres and hydrogen bonds as rows of small grey spheres. The side chains of residues involved in intermolecular contacts are shown and labelled.

The inter-domain linker is three residues longer in boophilin than in ornithodorin. Nevertheless, it runs closer to the proteinase surface in thrombin bound to the former inhibitor (Figure 7A). This linker peptide fits tighter to thrombin's surface features and engages in new interactions with thrombin not seen in the thrombin·ornithodorin complex, further contributing to inhibitor binding. The most relevant of these are hydrophobic/aromatic contacts of ^B^F80 with ^T^F34, as well as a water-mediated hydrogen bond between the carboxylate of ^B^E81 and the main chain nitrogen of ^T^Y76 (Figure 7B). (We use superscripts “B” and “T” before residue names to denote boophilin and thrombin residues, respectively).

Unexpectedly, the two Kunitz domains of boophilin not only diverge largely in three-dimensional structure from ornithodorin, but also occupy significantly different positions relative to the proteinase moiety, when compared to the thrombin·ornithodorin complex (Figure 7A). In particular, the N-terminal boophilin moiety rotates about nine degrees and is shifted ≈6 Å to the “left” in the standard orientation depicted in this figure. As for the C-terminal module, it is in boophilin rotated by approximately six degrees and translated by ≈3 Å “upwards”. These differences in quaternary arrangement of Kunitz modules relative to the serine proteinase moiety result in large differences when compared to the thrombin·ornithodorin complex, as we discuss below.

### Water-mediated interactions dominate at the interface between thrombin exosite I and boophilin

The C-terminal domain of boophilin engages in both hydrophobic and electrostatic interactions with thrombin's exosite I. In contrast to the thrombin·ornithodorin complex, however, where direct interactions between side chains of the proteinase and the inhibitor dominate at this interface, in the boophilin complex the equivalent residues are mostly involved in water-mediated interactions (Figure 7B). In addition, the guanidinium group of ^T^R77A donates hydrogen bonds to the hydroxyl group of ^B^Y129 and the carbonyl oxygen of ^B^D126. Further direct hydrogen bonds are formed between the carboxylate of ^B^E134 and the hydroxyl group of ^T^Y76. These polar contacts are strengthened by hydrophobic interactions between the side chains of ^B^L137 and a pocket formed by proteinase residues ^T^L65, ^T^R67 and ^T^I82, as well as between ^B^V138 and ^T^Y76.

We note that the exosite I-contacting face of boophilin, although displaying an overall negative electrostatic potential, harbours a smaller cluster of acidic residues than ornithodorin, which are located closer to the interdomain linker. Unexpectedly, none of the three salt bridges between glutamates in ornithodorin and the proteinase moiety are observed in the complex with boophilin, even though these acidic residues are conserved or conservatively replaced. Likewise, of the five residues involved in hydrophobic contacts in ornithodorin, one (I117) has no equivalent in boophilin, while the structural equivalents of two others (^B^Y129 for F103 and ^B^E133 for V107) have an increased polar character and are no longer involved in van-der-Waals interactions (Figure 7B). Although the total buried area upon thrombin complexation by boophilin is equivalent to that of the thrombin·ornithodorin complex (∼1,800 Å^2^), the decrease in the number and strength of the proteinase-inhibitor interactions offers an explanation for the lower *K*_i_ determined for boophilin (see Discussion).

It is noteworthy that a good fraction of boophilin's negative charges cluster in the solvent exposed side of the molecule, pointing away from the basic exosite I of thrombin (Supplementary Figure S2). This suggests that the C-terminal domain serves a multiple role, sterically blocking substrate access, masking the overall positive charge of the exosite, and creating a new negatively-charged surface that can repel approaching thrombin substrates. These combined effects would thus counteract competitive displacement of the inhibitor by physiological substrates, which require previous exosite I binding for processing.

### The N-terminal residues of boophilin occupy the active site cleft in a parallel manner

**Figure 8:**
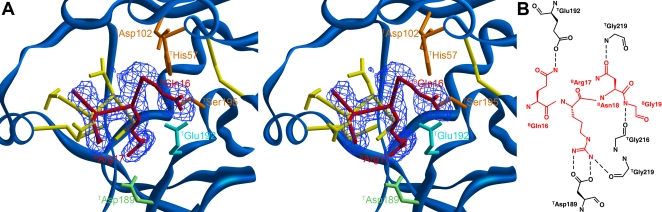
Boophilin inhibits thrombin in a non-canonical manner. (A) Stereo close-up of the thrombin active centre (blue) showing the bound tetrapeptide ^B^Q16–^B^R17–^B^N18–^B^G19 of boophilin (red), along with S1A–L1–N2–V3 of ornithodorin (yellow; notice that the N-terminal residue of the latter is an artifact introduced for cloning purposes). The final electron density for the thrombin·boophilin complex, contoured at 1σ, is displayed as a blue mesh. The catalytic triad of thrombin (^T^H57, ^T^D102, ^T^S195) is highlighted in orange and the side-chains of ^T^D189 and ^T^E192 are coloured pale green and cyan, respectively. Disulfide bonds are represented as yellow sticks. (B) Schematic representation of the thrombin-boophilin interactions at the enzyme's active site. Inhibitor residues are coloured red and hydrogen bonds are depicted as dashed lines.

The experimental electron-density map (Figure 8A) unambiguously shows that the N-terminal residues of boophilin bind across thrombin's active site cleft in a parallel, hirudin-like manner [16]. In the three-dimensional structure of the thrombin·ornithodorin complex, it was observed that the introduction of a non-natural serine residue at the N-terminus of the inhibitor results in an unfavourable chemical environment around the side chain of ^T^E192. By contrast, the present structure shows that this carboxylate group is stabilised by hydrogen-bonding interactions with the side chain of the natural N-terminal residue of mature boophilin, ^B^Q16 (see Figure 8B for a schematic representation of major thrombin-boophilin interactions). Inspection of the electron density maps suggests that the latter side chain displays a somewhat increased flexibility, which correlates well with its above-average B-factor. The main chain of this N-terminal residue is positioned in the S_2_ pocket of thrombin, stabilised by a direct hydrogen bond between its carbonyl oxygen and the guanidinium group of ^B^R22.

The most relevant feature of active site interactions is the occupancy of thrombin's S_1_ subsite by the ideally suited side chain of ^B^R17. The guanidinium group of this boophilin residue forms two hydrogen bonds with the carboxyl group of ^T^D189 (3.4 and 3.6 Å) at the bottom of the S_1_ pocket (Figure 8A). Two additional hydrogen bonds are formed between the terminal group of ^B^R17 and the main chain carbonyls of ^T^G219 (direct bond) and ^T^F227 (water-mediated). This feature also distinguishes boophilin from ornithodorin, which lacks an equivalent residue at this position.

The following boophilin residue, ^B^N18, interacts through its side chain with the main chain nitrogen atom of ^T^G219 and the carboxyl oxygen of ^T^E146, the latter via a solvent molecule. Finally, ^B^G19 is hydrogen-bonded to the carbonyl group of ^T^G216 (Figure 8B), thus partially mimicking the interaction of a ligand occupying the S_3_ pocket of the proteinase. This subsite accommodates the side chain of ^B^R22, which forms a direct hydrogen bond with the hydroxyl group of ^T^Y60A. The only other major contact between the proteinase moiety and the N-terminal domain of boophilin is the parallel stacking of the side-chain of ^B^F39 with those of ^T^P60C and ^T^W60D.

## Discussion

### Tick-derived anti-hemostatics

We report the isolation and cloning of a two-domain Kunitz inhibitor from the cattle tick *Boophilus microplus*, which we have termed boophilin. Further, we characterise boophilin's inhibitory mechanism by solving the three-dimensional structure of its complex with bovine thrombin. Together with our identification of carrapatin, a single domain Kunitz-type inhibitor in engorged ticks (Swiss-Prot accession number P81162), as well as with previous reports of inhibitors isolated from eggs and larvae of this parasite [28]–[34], [40], evidence is emerging that *B. microplus* utilizes a whole array of both single- and multi-domain Kunitz inhibitors during its whole life cycle. Recently, several salivary proteins that contain single or tandem Kunitz repeats have also been identified in the hard ticks *Ixodes scapularis* [41] and *I. pacificus* [42], indicating that inhibitors from this family might play a general role as antihemostatic factors. One of these proteins, the double-headed inhibitor, ixolaris, was shown to inhibit the tissue factor·FVIIa complex in a FX(a)-dependent manner by binding to exosite II in FXa, in line with its homology to TFPI [43], [44].

Recently, another two-domain Kunitz inhibitor with anti-thrombin activity, amblin from the hard tick *Amblyomma hebraeum*, has been described [37]. Notably, amblin differs from boophilin due to (i) a much shorter interdomain linker; (ii) a relatively long, highly basic C-terminal extension; (iii) the presence of two additional cysteine residues located in this extension and in the C-terminal Kunitz domain, respectively, which might form an extra disulfide bond; and (iv) non-conservative replacements of several acidic residues of the C-terminal domain (Figure 3). We have generated a three-dimensional model of amblin on the basis of its closest relative of known structure, bikunin [45]. The high pI of amblin is reflected in extended areas of basic potential on both Kunitz domains (Supplementary Figure S2). Thus, both strong electrostatic repulsion and inappropriate quaternary structure would make extremely unlikely that amblin interacts with any of the positively charged exosites on the thrombin moiety. The lack of anti-trypsin activity by amblin is also surprising in view of the conservation of “canonical” reactive-site loops, and because our homology model suggests that the reactive-site loop of the N-terminal Kunitz domain remains accessible to target proteinases. These observations require a thorough investigation of amblin's inhibitory potential.

### Boophilin is a canonical Kunitz-type inhibitor

Inhibition of the key procoagulant enzyme, thrombin, by canonically binding Kunitz-type inhibitors is unfavourable due to the presence of a prominent insertion loop at position 60, and of a glutamate at position 192. These obstacles are circumvented in the double-headed Kunitz inhibitor from *O. moubata*, ornithodorin, as only its N-terminal peptide inserts into the active-site cleft of the proteinase [25]. A highly similar thrombin inhibitor, savignin, has been isolated from the salivary glands of *O. savignyi* [46], [47], and would be expected to use the same non-canonical mechanism for thrombin inhibition. On the other hand, the close resemblance of boophilin to canonical Kunitz domains and the presence of an optimal P_1_ residue in its N-terminal domain (K31) suggested that boophilin could inhibit thrombin in a canonical manner, i.e. by occupying the active-site cleft of the proteinase with the reactive-site loop of this module. This would require a considerable repositioning of the N-terminal Kunitz module compared to the thrombin·ornithodorin complex, which is nevertheless possible given that the two domains are connected by a long, flexible linker. Canonical binding would in turn require that initial binding of the C-terminal domain to exosite I provided enough energy to drive insertion of the N-terminal reactive site loop past the energetic barrier that thrombin's 60-loop represents.

### Boophilin inhibits thrombin in a non-canonical manner, despite possessing a canonical reactive-site loop

However, structural and functional evidence presented here indicates that boophilin also adopts the non-canonical mechanism for thrombin inhibition, in spite of possessing a regular BPTI-like domain. We conclude that binding of the acidic C-terminal Kunitz domains to exosite I does not induce major rearrangements of loops surrounding thrombin's active-site cleft. This is in line with our previous observations of essentially unmodified active sites in the crystal structures of thrombin·triabin [15] and thrombin·thrombomodulin [24] complexes. A recent crystal structure of murine thrombin bound to an acidic peptide from PAR3 via exosite I revealed some displacements of 60-loop residues, in particular W60D [48]. However, analysis of the thrombin·PAR3 complex (PDB entry 2PUX) indicates that these rearrangements are either induced or stabilised by artifactual interactions with a neighbouring crystal molecule. In conclusion, it would seem that interactions with exosite I neither induce rearrangements of thrombin structure, in particular of the 60-loop, nor are required for inhibitors or substrates to approach the proteinase catalytic machinery. Exosite-mediated interactions are nevertheless strictly required for positioning unfavourable substrates (protein C, thrombin-activatable fibrinolysis inhibitor/TAFI)/inhibitors (Kunitz) at a proper distance and orientation to gain access to the active site of thrombin.

In spite of similar domain organisations and the presence of highly acidic C-terminal domains, ornithodorin/savignin are much tighter thrombin inhibitors (*K*_i_ = 1.0×10^−12^ M or 4.9×10^−12^ M) than boophilin (*K*_i_ = 1.8×10^−9^ M). Further, whereas ornithodorin and savignin do not cross-react with other serine proteinases, boophilin is a potent inhibitor of trypsin and of the fibrinolytic enzyme, plasmin. It is tempting to speculate that the less efficient thrombin inhibitor, boophilin, confers an evolutionary advantage to *B. microplus* by controlling opposing mechanisms of hemostatic balance. Another interesting possibility is discussed below. Of note, a thrombin inhibitor with an apparent M_r_ ∼60 kDa, BmAP, has been isolated from the saliva of *B. microplus* [49]. In contrast to boophilin, BmAP does not inhibit plasmin or trypsin. Finally, a small (M_r_ = 1,770 Da) exosite-binding thrombin inhibitor, microphilin, has been identified in the tick saliva [50]. It is thus conceivable that the three inhibitors cooperate to inhibit thrombin activity during feeding and digestion.

It is also worth mentioning that immunization of cows with salivary gland preparations from *B. microplus* reduced babesiosis [51], pointing to the immunological potential of proteins secreted by this parasite. Of note, the sequence containing the reactive-site loop in boophilin N-terminal domain is predicted to be the major antigenic determinant of the protein. Given that thrombin inhibitors are critical for hematophagous animals during feeding and digestion, boophilin would appear as a promising candidate for the development of recombinant vaccines.

The close relatedness of boophilin to BPTI-like Kunitz inhibitors (Figures 3 and 6) explains its ability to potently inhibit trypsin and plasmin. In particular the N-terminal domain possesses all the necessary features for canonical inhibition, including a lysine side chain (K31) ideally suited for occupying the S_1_ pocket of trypsin-like serine proteinases with a serine at position 190 [7], [52], [53]. Further, the I29 side chain is preferred at the P_3_ position in Kunitz-type inhibitors [53]. Finally, the conserved alanine residue at position P_1_' is also essential for formation of stable complexes with trypsin and plasmin [54]. For several other serine proteinases tested, the presence of A190 at the bottom of the S_1_ specificity pocket, instead of a serine in e.g. trypsin and plasmin, increases the size of this pocket and eliminates a potential hydrogen-bond partner for a lysine side chain [55]. In these cases, Kunitz inhibitors with an arginine at position P_1_ are strongly preferred over those with a P_1_ lysine [52], [53], thus explaining at least partially the poor or absent effect of boophilin on FXa, FXIIa, uPA and t-PA. Finally, in the tryptase tetramer the active sites of all four monomers are arranged facing an internal pore, and are therefore inaccessible to most substrates and inhibitors [56]. Kunitz inhibitors are too bulky to enter the tryptase channel, explaining the inactivity of boophilin towards tryptase.

### Boophilin is a bi-functional serine proteinase inhibitor

**Figure 9:**
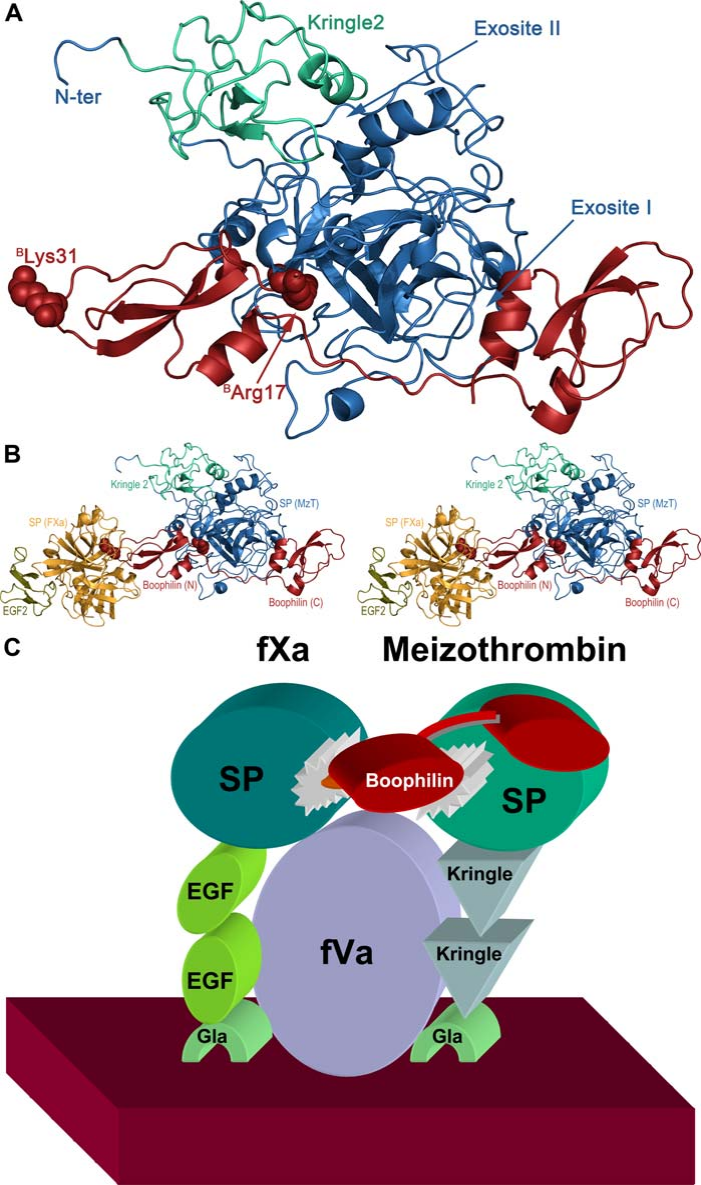
Predicted boophilin interactions with meizothrombin and FXa. (A) Model of the three-dimensional meizothrombin·boophilin complex, generated by overlaying the coordinates of bovine meizothrombin (blue except for the Kringle 2 domain, in green; 1A0H) [57] onto the thrombin moiety of the current complex with boophilin. Residues ^B^R17 and ^B^K31 are shown as space-filling models and labelled. (B) Model of the putative MzT·boophilin·FXa ternary complex. FXa (yellow, except for domain EGF2, in olive; 1FJS) [58] was docked onto the complex displayed in (A) by juxtaposition onto a trypsin·APP complex (1TFX) [6] that had been previously overlaid onto the N-terminal domain of boophilin (red). Absence of any steric clashes shows that formation of such a ternary complex *in vivo* would be feasible. The N- and C-terminal domains of boophilin (Boophilin (N) and Boophilin (C), respectively), the serine proteinase and Kringle 2 domains of meizothrombin (SP (MzT) and Kringle 2, respectively), and the serine proteinase and EGF-2 domains of factor Xa (SP (FXa) and EGF-2, respectively) are labelled. (C) Schematic representation of a possible mechanism for boophilin inhibition of the membrane-bound prothrombinase complex. Both FXa and meizothrombin interact with the membrane surface (dark red) via their respective Gla domains (light green). The multi-domain organisation of FVa (violet) and its contacts with FXa/MzT have been omitted for simplicity. The N-terminal domain of boophilin (red) could bridge the catalytic domains of meizothrombin (dark green) and FXa (blue) while bound to the cofactor.

As mentioned above, boophilin possesses a rather high *K*_i_ for thrombin inhibition when compared to the two other thrombin inhibitors known or predicted to bind in a non-canonical manner, ornithodorin [25] and savignin [46], [47]. This observation raises the interesting possibility that boophilin could have evolved to target not only circulating thrombin, but to block in addition the membrane-bound activation intermediate, meizothrombin (MzT). The crystal structure of a MzT variant that lacks the membrane-binding fragment 1, reveals formed exosite I and active centre, while exosite II is covered by the kringle 2 domain of the intermediate form [57]. Therefore boophilin could dock to this form essentially as seen in the current crystal structure (see Figure 9A for a representation of the modelled MzT·boophilin complex).

Our current data also indicate that thrombin-bound boophilin retains the capability to interact with additional serine proteinases, as demonstrated by formation of a stable ternary complex with trypsin (Figure 4), and the reactive-site loop of the N-terminal boophilin domain would remain fully accessible in the MzT·boophilin complex (compare Figures 5 and 9A). These observations immediately suggest that a proximal, membrane-bound proteinase could easily dock to this complex. Coagulation FXa would appear as the most attractive candidate, because of its proximity to MzT during prothrombin activation by the prothrombinase complex (FVa·FXa). To verify that MzT·boophilin·FXa ternary complex formation is feasible without steric clashes, we overlaid the structurally and functionally related second Kunitz domain of TFPI (refer to Figure 6A) bound to trypsin [6] onto the N-terminal boophilin domain. Next, we superposed the FXa crystallographic model [58] onto the trypsin moiety. This revealed that the serine proteinase moieties of FXa and meizothrombin could be cross-linked by the N-terminal domain of a boophilin molecule (Figure 9B). Furthermore, the N-terminal domains of both proteinases would not interfere with ternary complex formation. In this manner, boophilin could efficiently inhibit thrombin generation by prothrombinase. A schematic representation of this putative, membrane-bound complex is shown in Figure 9C. We stress, however, that free boophilin only marginally inhibits FXa *in vitro* (Table 1), and thus formation of the ternary complex would rely on the proper orientation of both membrane-bound serine proteinases, and would probably require additional boophilin-FVa interactions. In support of our hypothesis, we note that both endogenous (TFPI) [4] and tick-derived multidomain Kunitz inhibitors ixolaris [43], [44] and penthalaris [59] employ this dual strategy for inhibiting the related FVIIa·TF·FXa complex. Of particular note, TFPI inhibits FVIIa through its first Kunitz domain, but only after the second Kunitz moiety has bound FXa [4], [60]. Future investigations should test the validity of this model, or identify other targets for thrombin-bound boophilin *in vivo*.

Finally, we notice that the current structure has implications for modelling procedures, in particular for macromolecular docking. Current docking algorithms have only had limited success rates, even when the structures of isolated modules were known [61]. Our structure of the thrombin·boophilin complex and its comparison with the related thrombin·ornithodorin heterodimer reveals that caution must be exercised even when a highly homologous template for the expected complex does exist. Indeed, these two inhibitors share a common binding mode (blockade of basic exosite I of the proteinase and parallel alignment of N-terminal residues across the active-site cleft). However, the details of interacting residues (e.g., direct vs. water-mediated contacts at exosite I, occupancy or not of the major S_1_ subsite on thrombin) vary considerably in their complexes with the proteinase. Concomitantly, and perhaps more striking from the viewpoint of macromolecular docking, the two pairs of inhibitor domains adopt markedly different orientations relative to the cognate thrombin molecule.

## Materials and Methods

### Animals

Ticks were obtained from Drs. D.H. Aguirre and A.B. Gaido, Instituto Nacional de Tecnología Agropecuaria, Estación Experimental Agropecuaria Salta (Argentina).

### Reagents

Bovine α-thrombin was isolated from fresh ox blood as described previously [62]. Other serine proteinases were purchased from the suppliers listed below: bovine FXa, Diagnostic Reagents Ltd. (Oxon, England); human α-FXIIa and plasma kallikrein, Kordia Laboratory Supplies (Leiden, Netherlands); recombinant human FVIIa (NovoSeven®), Novo Nordisk (Denmark); human plasmin, Behringwerke GmbH (Marburg, Germany); recombinant tryptase, Promega (Madison, USA); recombinant sc-tPA, Boehringer Mannheim (Mannheim, Germany); bovine pancreatic trypsin, Serva Feinbiochemika (Heidelberg, Germany); and urokinase (uPA), Ribosepharm GmbH (Haan, Germany). All chromogenic substrates used were obtained from Pentapharm AG (Basel, Switzerland), except for Chromozym® TH (Tos-Gly-Pro-Arg-para-nitroanilide), purchased from Boehringer Mannheim, which also provided APTT reagent and endoproteinases Lys-C and Asp-N. Dade® Thromboplastin-IS was purchased from Dade Behring Marburg (Marburg, Germany). All other chemicals, of the highest purity grade available, were purchased from Merck (Darmstadt, Germany), except where noted.

### Isolation of native boophilin

One-hundred fifty grams of frozen engorged ticks were homogenised in 1 l buffer A (20 mM Tris-HCl, pH 8.0, 1 mM CaCl_2_, 1 mM benzamidine). After centrifugation at 10,000×g for 10 minutes, the filtered (5-µm pore) supernatant was applied to a Q-Sepharose (Pharmacia, Freiburg, Germany) column (200×50 mm) previously equilibrated with 20 mM Tris-HCl, pH 8.0 (buffer B). The column was extensively washed with buffer B and bound proteins were eluted with a linear NaCl gradient (from 0 to 1.0 M in buffer B).

A thrombin affinity column was prepared by coupling 10 mg of bovine α-thrombin to 2 g of CNBr-activated Sepharose 4B (Pharmacia), according to the manufacturer's guidelines. Ion-exchange chromatography fractions with thrombin inhibitory activity (determined as described below) were pooled, diluted with two volumes of 20 mM sodium phosphate, pH 7.4 (buffer C) and applied to the thrombin-Sepharose column previously equilibrated in buffer C. The column was extensively washed with buffer C and subsequently with buffer C containing 500 mM NaCl and with double distilled water, before elution of specifically bound proteins with 20 mM glycine-HCl, pH 2.5. The pH of the eluate was immediately adjusted to 8.0 with 0.1 volumes 1 M Tris-HCl, pH 8.0 before lyophilising the sample.

### Activity assays

Thrombin inhibitory activity was followed during boophilin purification by measuring the residual amidolytic activity of the proteinase towards Chromozym® TH. Bovine thrombin (900 µl, 15 nM) in assay buffer (50 mM Tris-HCl, pH 7.8, 0.1 M NaCl, 0.05% PEG 6,000) was incubated with 50 µl protein samples or with the same volume of assay buffer for 2 minutes at 37°C. Substrate (50 µl, 1.5 mM) was then added, and absorbance of the reaction mixture at 405 nm was monitored for 2 minutes.

### Inhibition of blood clotting by boophilin

To determine thrombin time, 100 µl citrated human plasma were mixed with 50 µl 0.154 M NaCl or inhibitor solution and equilibrated at 37°C. Bovine thrombin (50 µl; 5 IU/ml) was then added and the time to clot was measured. Prothrombin time was determined by mixing 50 µl of Dade® Thromboplastin-IS with 50 µl of either 25 mM CaCl_2_ or the same solution containing the sample to be assayed. After equilibration at 37°C, 50 µl human citrated plasma were added, and the coagulation time measured. Activated partial thromboplastin time was determined both with and without preincubation of the inhibitor solution (40 µl) with 50 µl citrated human plasma and 50 µl APTT reagent for 3 minutes at 37°C. Ten µl of a 125 mM CaCl_2_ solution were finally added and the coagulation time was measured. Clotting times were determined in duplicate.

### Inhibition of other serine proteinases

The amidolytic activity of each proteinase towards a specific chromogenic substrate was assayed at room temperature, by measuring the variation of light absorbance at 405 nm in the assay mixture. Briefly, 200 µl of assay buffer (50 mM Tris-HCl, pH 7.4–8.4, 154 mM NaCl) or of inhibitor were mixed with 50 µl of the enzyme solution and dispensed in 96-well microtiter plates. After five minutes pre-incubation, 25 µl of the chromogenic substrate solution were added and the reaction was allowed to proceed for an enzyme-dependent period (2–6 minutes). Reactions were stopped by adding 25 µl 50% (v/v) acetic acid. The enzymes tested are given in the legend to Table 1, along with the chromogenic substrates used in each case.

For determination of the second-order rate constants of trypsin/plasmin inhibition, 200 µl of inhibitor solution were mixed with 25 µl substrate (Chromozym TH or Chromozym PL, respectively; final concentration: 0.2 mM). The final concentration of recombinant boophilin was varied between 0.1 and 0.65 µM (trypsin), or between 0.04 and 0.4 µM (plasmin). The reaction was started by addition of 25 µl enzyme solution (final concentrations: 6.25 ng/ml and 12.5 µg/ml, respectively), and the absorption at 405 nm was measured over 45 minutes at 37°C. The pseudo-first order rate of inactivation for each inhibitor concentration (k_obs_) was determined from the progression curve according to the equation [P] = A+Bt–Cexp(-k_obs_t). Second-order rate constants were calculated from the equation k_2_/*K*_i_ = (1+[S_0_]/K_m_)/b, with S_0_ = 0.2 mM, *K*_m_ = 0.0117 mM for Chromozym® TH or 0.286 mM for Chromozym® PL; the slope b was computed from the regression line 1/k_obs_ *versus* 1/[I].

### Electrophoretic mobility shift assays

Five-hundred ng human thrombin or bovine trypsin in 25 mM Tris-HCl pH 8.0, 190 mM glycine, were mixed with increasing concentrations of boophilin and incubated at room temperature for 15 min before adding glycerol to 5% final concentration. In other experiments, thrombin·boophilin complexes were similarly titrated with increasing concentrations of trypsin. Samples were resolved at 4°C in Tris-glycine-polyacrylamide gels and silver-stained.

### Protein sequencing

Lyophilised protein samples were dissolved in 0.1 M Tris-HCl, pH 8.0, 6 M guanidinium hydrochloride, 5% (v/v) β-mercaptoethanol and incubated for 16 hours at room temperature to reduce disulfide bonds. The free thiol groups were derivatised with 5% (v/v) 4-vinylpyridine; samples were incubated for further 90 minutes at room temperature, acidified with formic acid, and finally desalted by RP-HPLC. The S-β-pyridylethylated samples were digested for 10 hours at 37°C with either Lys-C (in 25 mM Tris-HCl, pH 8.5, 1 mM EDTA) or Asp-N (in 50 mM sodium phosphate, pH 8.0). Reactions were stopped by adjusting the pH of the samples to 2.0 with formic acid. For CNBr-cleavage, S-β-pyridylethylated boophilin was dissolved in 70% (v/v) formic acid containing 10% (w/v) CNBr and incubated in the dark at room temperature for 14 h. Peptides were separated by RP-HPLC and subjected to automated sequencing. The molecular mass of the unmodified protein was determined by MALDI-MS.

### Construction and screening of the *B. microplus* cDNA library

Total RNA was isolated from 1 g of unfed ticks (for cDNA library construction) or from 10 g of engorged adults (for PCR amplification) and enriched in poly(A)^+^ RNA by oligo(dT)-cellulose affinity chromatography. cDNA was synthesised from 1 µg of poly(A)^+^ RNA with random hexanucleotides as primers, and a cDNA library was constructed in λ Uni-ZAP XR (Stratagene, Amsterdam, Netherlands), following the manufacturer's instructions.

An internal fragment of boophilin cDNA was amplified by PCR using degenerate oligonucleotides (Supplementary Table S2), cloned into the *BamH* I site of pBluescript KS (Stratagene, Amsterdam, Netherlands), and sequenced. A fragment of similar size was amplified using aliquots of the cDNA library phage stock as template, to verify the presence of boophilin cDNA in the library. Positive clones, identified using the α-^32^P-dATP-labelled boophilin cDNA fragment as probe, were isolated and converted into double stranded pBluescript phagemids by *in vivo* excision, and further characterised by automated DNA sequencing of both strands.

### Heterologous expression of mature boophilin

Mature boophilin (variant H2) was cloned into the bi-cistronic expression vector pRBI-DsbC [63] by overlap-extension PCR. Briefly, the OmpA signal-coding region of the vector and the fragment of boophilin cDNA coding for the mature inhibitor were amplified independently, using specific primers (Supplementary Table S2). Aliquots of both PCR products were then mixed and amplified using the previously used 5′ primer for the OmpA fragment and the 3′ primer for boophilin. The amplified fragment, coding for an OmpA signal–mature boophilin fusion protein was cloned into the *Xba* I and *Hind* III sites of the expression plasmid.

*Escherichia coli* DH5α cells transformed with the expression construct were grown in LB with 2 g/l glucose and 100 mg/l ampicillin to an optical density of 0.7, before induction with 1 mM IPTG (isopropyl-β-D-thiogalactopyranoside). At induction time, 2 g/l glucose, 100 mg/l ampicillin, and 5 mM *N*-acetyl-cysteine were added. Cells were harvested 36 hours post-induction and periplasmic proteins were released *via* cold osmotic shock. The resulting solution was immediately applied to an 80×50 mm Q-Sepharose FF column pre-equilibrated with buffer D (20 mM Tris-HCl, pH 8.0). The column was extensively washed with buffer D, and bound proteins eluted with a linear salt gradient (from 0 to 0.5 M NaCl in buffer D). Fractions displaying anti-thrombin activity were pooled, diluted with buffer D and loaded onto a ResourceQ column (Pharmacia, Freiburg, Germany) pre-equilibrated with the same buffer. The column was extensively washed with buffer D and bound proteins were eluted with a linear salt gradient (0–0.4 M NaCl in buffer D). Samples with anti-thrombin activity eluted as a single peak. Correct processing of the recombinant inhibitor was verified by MALDI-MS and N-terminal sequencing.

### Complex preparation and crystallisation

The macromolecular complex was prepared by mixing bovine α-thrombin with a molar excess of purified recombinant boophilin. The mixture was incubated on ice for two hours, and the thrombin·boophilin complex was separated from excess inhibitor by size exclusion chromatography on a Superdex 75 10/30 column (Amersham Pharmacia), using 0.01 M MES pH 6.5, 0.1 M NaCl as the mobile phase. Fractions were collected and analysed by SDS-PAGE. Those containing the thrombin·boophilin complex were pooled and concentrated on a 10-kDa cut-off centrifugal concentrator (Vivascience).

Single crystals of the macromolecular complex were obtained by vapour diffusion at 279 K from 4-µl sitting drops containing equal volumes of bovine thrombin·boophilin complex solution (7.5 mg/ml in 0.01 M MES pH 6.5, 0.1 M NaCl) and precipitant (0.05 M KH_2_PO_4_, 18% (w/v) PEG 8,000). Crystals belong to the orthorhombic space group P2_1_2_1_2_1_, with unit cell constants a = 92.5 Å, b = 104.2 Å, c = 129.1 Å, and contain two complexes per asymmetric unit (solvent content 60.6%). The crystals were transferred to 1.5× mother liquor containing 15% glycerol as cryoprotectant and cryo-cooled in liquid nitrogen.

### Data collection, processing and refinement

Diffraction data to 2.35 Å were collected from a single cryo-cooled (100 K) crystal on a MAR CCD detector at ESRF beamline ID14-EH3. Data were processed with MOSFLM [64], scaled with SCALA [65], and reduced with tools from the CCP4 package [65]. Data collection and refinement statistics are summarised in Table 2.

**Table 2:** Statistics of data collection and refinement

Crystallographic analysis	
Resolution range (Å) (overall/outer shell)	54.9–2.35/2.48–2.35
Space group	P2_1_2_1_2_1_
Unit cell dimensions (Å)	a = 92.5 b = 104.2 c = 129.2
Number of observations (total/unique)	192,392/51,554
Multiplicity (overall/outer shell)	3.7/3.7
R_merge_ a (overall/outer shell)	6.4/29.0
Completeness (%) (overall/outer shell)	98.0/97.4
I/σ(I) (overall/outer shell)	9.5/2.6
Mathews coefficient (Å^3^ Da^−1^)	3.1
Solvent content (%)	60.6
**Structure refinement**	
R_factor_ b/Free R_factor_ c (%)	19.0/23.0
N° of unique reflections (working/test set)	48,886/2,627
Water molecules	453
Ions	6 (4 PO_4_^3−^, 2 Na^+^)
Total number of atoms	7,261
Number of protein atoms	6,771
Average overall B-factor (Å^2^)	31.2
Average protein B-factor (Å^2^)	30.8
Average main-chain B-factor (Å^2^)	30.5
Average side-chain B-factor (Å^2^)	31.2
Average water B-factor (Å^2^)	33.1
Average ion B-factor (Å^2^)	62.4 (PO_4_^3−^)/32.5 (Na^+^)
r.m.s.d. bonded B́s (Å^2^)	0.751
r.m.s.d. bond lengths (Å)	0.008
r.m.s.d. bond angles (°)	1.137
**Ramachandran plot statistics**	
Residues in allowed regions	717 (99.9%)
Residues in generously allowed regions	1 (0.1%)
Residues in disallowed regions	0 (0%)
**Estimated coordinate error**	
E.s.d. from Luzzati plot (Å)	0.30
DPI^d^ (Å)	0.20

a R_merge_ =  Σ_h_Σ_i_ |I_hi_-<I_h_>|/Σ_h _Σ_i _<I_h_>, where I_hi_ is the observed intensity of the i-th measurement of reflection (h), including symmetry-related ones, and <I_h_> is the mean intensity of the i observations of reflection h over all measurements of I_hi_.

b R_factor_ = Σ||F_o_|-|F_c_||/Σ |F_o_| where |F_o_| and |F_c_| are observed and calculated structure factor amplitudes, respectively.

c Free R_factor_ is the cross-validation R-factor computed for a randomly chosen subset of 5% of the total number of reflections, which were not used during refinement.

d Diffraction-data precision indicator

The structure was solved by molecular replacement using AMoRe [66] with data in the 10-4.0 Å resolution range. Two proteinase molecules were located using the coordinates of unliganded bovine α-thrombin from PDB entry 1MKX [67] as search model. All attempts to locate the Kunitz domains of boophilin by molecular replacement, using truncated models of either BPTI (1BPI) [68] or ornithodorin (1TOC) [25] were unsuccessful.

The initial electron density maps, computed after rigid body refinement of the proteinase molecules with CNS v. 1.1 [69], displayed additional density for most of the inhibitor molecules. Two cycles of manual rebuilding of the proteinase models on a graphic workstation using TurboFRODO (Bio-Graphics, France), followed by positional refinement with CNS resulted in significant improvement of the electron density maps. This allowed manual docking of a truncated model of BPTI close to thrombin exosite I for each of the complexes. Further crystallographic refinement alternated with manual model building until complete interpretation of the electron density maps. The final isotropically refined model obtained with CNS was further refined with REFMAC_5 [70] using TLS displacement parameters.

The final model comprises residues ^T^F1P to ^T^R15 and ^T^I16 to ^T^D243 of one thrombin molecule (chains A and B, respectively) and residues ^T^P1N to ^T^E14L and ^T^I16 to ^T^D243 of the other proteinase moiety (chains C and D, respectively). Both boophilin molecules (chains E and F) were modelled from ^B^Q16 to ^B^M142. The side chains of^ T^E14L (chain A), ^T^K236 (chain B), ^T^K1I, ^T^K14D (chain C), ^T^K87, ^T^R93, ^T^K97, ^T^E113 (chain D), ^B^E75, ^B^E110 (chain E), and of ^B^K86 (chain F) are not well defined in the electron density maps; we have assigned an occupancy value of zero to these side chains. The model comprises also three phosphate and two sodium ions, 453 water molecules and an N-acetyl-glucosamine sugar moiety attached to ^T^N60G in chain D.

### Modelling and other computational methods

Sequence similarity searches were performed with the program FastA from the Wisconsin package (version 9.0, Genetics Computer Group, Madison, Wisconsin), and alignments were prepared with ALSCRIPT [71]. Major antigenic peptides were predicted with the Kolaskar & Tongaonkar method (http://bio.dfci.harvard.edu/Tools/antigenic.pl). Models of the thrombin·boophilin complex were built based on the co-ordinates of (E192Q)thrombin·BPTI (1BTH) [23] and thrombin·ornithodorin (1TOC) [25] complexes, and were subjected to energy minimization with CNS. The amblin model was built by applying homology-based methods as implemented in the automatic SWISS-MODEL server (http://swissmodel.expasy.org) [72], using the structure of the Kunitz inhibitor bikunin as template [45]. Structure representations were generated with PyMOL (http://www.pymol.org) or SETOR [73].

### Accession codes

The complete nucleotide sequences of both boophilin isoforms have been deposited with the EMBL database under accession numbers **AJ304446** and **AJ304447**, respectively. Coordinates for the bovine α-thrombin·boophilin complex have been deposited with Protein Data Bank, accession number **2ODY**.

## Supporting Information

Figure S1 Native boophilin is a small disulfide-linked, acidic thrombin inhibitor. Mass spectrum of affinity-purified native boophilin obtained using MALDI-TOF. Notice the position of the myoglobin marker (both M+H^+^ and M+2H^+^ peaks). The inset shows the electrophoretic analysis of purified boophilin separated in a 15% SDS-polyacrylamide gel, stained with Coomassie Brilliant Blue. Lane 1, non-reduced boophilin sample; lane 2, molecular mass marker; lane 3, reduced boophilin; molecular mass markers are given to the right.
(0.82 MB TIF)

Figure S2 Two-domain Kunitz inhibitors of thrombin display different surface potentials. Electrostatic surface potential (red, negative; blue, positive) representation of (A) boophilin, (B) ornithodorin, and (C) amblin. For image pairs in panels (A) and (B) the left image corresponds to the exposed side of the molecule when bound to the proteinase, and the right image results from a 180{degree sign} rotation around the y-axis, to expose the thrombin-binding surfaces to the viewer. The model of amblin is represented with its N-terminal domain in a similar orientation to those of the other inhibitors. Selected topologically equivalent residues in boophilin and ornithodorin are labelled.
(3.19 MB TIF)

Table S1 Amino acid sequences of peptides derived from purified native boophilin. Reduced and S-β-pyridylethylated samples were digested with endopeptidases Lys-C or Asp-N, or chemically hydrolysed with CNBr. The resulting fragments were separated by RP-HPLC and major peaks were sequenced; peptides obtained by either procedure are numbered according to their elution order in the corresponding chromatograms. Positions at which two different amino acid residues have been obtained are underlined; they indicate the existence of at least two boophilin isoforms. Overlapping peptides *CNBr-Asp-N4-(Lys-C5/Lys-C6)-Asp-N2-Lys-C15-Asp-N7* define a single sequence of 77 residues: FSYGGC^4^GGNENNFETIEDC^5^QKAC^6^GAPERV(N/S)DFE(S/G)ADFKTGC^1'^EPAADSGSC^2'^AGQLERWFYNVRSGEC^3'^ETFVYGGC^4'^ (S/G)GN. Based on the homology to BPTI, peptide *Lys-C11* appears to correspond to the segment between the second and the third cysteine residue of the N-terminal domain, i.e. preceding the assembled sequence, while *Asp-N8* could be assigned to the region immediately following the C-terminal residue in the assembled sequence.
(0.03 MB DOC)

Table S2 Oligonucleotides used for boophilin cloning. The *BamH* I (primers *Lys-C15Fwd* and *Lys-C15Rev*), *Xba* I (*OmpA-Fwd*) and *Hind* III sites (*Booph-Rev*) introduced for cloning purposes are underlined. R, Y, and N, represent equimolar amounts of A and G, C and T, and of all four bases, respectively. The triplet coding for Q16 in primer *Booph-Fwd* and the termination codon in *Booph-Rev* are given in bold letters.
(0.03 MB DOC)

## References

[1] Bork P, Downing AK, Kieffer B, Campbell ID (1996). Structure and distribution of modules in extracellular proteins.. Q Rev Biophys.

[2] Laskowski M, Kato I (1980). Protein inhibitors of proteinases.. Annu Rev Biochem.

[3] Ascenzi P, Bocedi A, Bolognesi M, Spallarossa A, Coletta M (2003). The bovine basic pancreatic trypsin inhibitor (Kunitz inhibitor): a milestone protein.. Curr Protein Pept Sci.

[4] Broze GJ (1995). Tissue factor pathway inhibitor and the revised theory of coagulation.. Annu Rev Med.

[5] Huber R, Kukla D, Bode W, Schwager P, Bartels K (1974). Structure of the complex formed by bovine trypsin and bovine pancreatic trypsin inhibitor. II. Crystallographic refinement at 1.9 Å resolution.. J Mol Biol.

[6] Burgering MJ, Orbons LP, van der Doelen A, Mulders J, Theunissen HJ (1997). The second Kunitz domain of human tissue factor pathway inhibitor: cloning, structure determination and interaction with factor Xa.. J Mol Biol.

[7] Helland R, Otlewski J, Sundheim O, Dadlez M, Smalas AO (1999). The crystal structures of the complexes between bovine beta-trypsin and ten P1 variants of BPTI.. J Mol Biol.

[8] Scheidig AJ, Hynes TR, Pelletier LA, Wells JA, Kossiakoff AA (1997). Crystal structures of bovine chymotrypsin and trypsin complexed to the inhibitor domain of Alzheimer's amyloid beta-protein precursor (APPI) and basic pancreatic trypsin inhibitor (BPTI): engineering of inhibitors with altered specificities.. Protein Sci.

[9] Schmidt AE, Chand HS, Cascio D, Kisiel W, Bajaj SP (2005). Crystal structure of Kunitz domain 1 (KD1) of tissue factor pathway inhibitor-2 in complex with trypsin. Implications for KD1 specificity of inhibition.. J Biol Chem.

[10] Zhang E, St. Charles R, Tulinsky A (1999). Structure of extracellular tissue factor complexed with factor VIIa inhibited with a BPTI mutant.. J Mol Biol.

[11] Bode W, Huber R (2000). Structural basis of the endoproteinase-protein inhibitor interaction.. Biochim Biophys Acta.

[12] Davie EW, Fujikawa K, Kisiel W (1991). The coagulation cascade: initiation, maintenance, and regulation.. Biochemistry.

[13] Bock PE, Panizzi P, Verhamme IMA (2007). Exosites in the substrate specificity of blood coagulation reactions.. J Thromb Haemost.

[14] van de Locht A, Lamba D, Bauer M, Huber R, Friedrich T (1995). Two heads are better than one: crystal structure of the insect derived double domain Kazal inhibitor rhodniin in complex with thrombin.. EMBO J.

[15] Fuentes-Prior P, Noeske-Jungblut C, Donner P, Schleuning WD, Huber R (1997). Structure of the thrombin complex with triabin, a lipocalin-like exosite-binding inhibitor derived from a triatomine bug.. Proc Natl Acad Sci USA.

[16] Rydel TJ, Ravichandran KG, Tulinsky A, Bode W, Huber R (1990). The structure of a complex of recombinant hirudin and human alpha-thrombin.. Science.

[17] Richardson JL, Kroger B, Hoeffken W, Sadler JE, Pereira P (2000). Crystal structure of the human alpha-thrombin-haemadin complex: an exosite II-binding inhibitor.. EMBO J.

[18] Ascenzi P, Coletta M, Amiconi G, de Cristofaro R, Bolognesi M (1988). Binding of the bovine basic pancreatic trypsin inhibitor (Kunitz) to human alpha-, beta- and gamma-thrombin; a kinetic and thermodynamic study.. Biochim Biophys Acta.

[19] Pintigny D, Dachary-Prigent J (1992). Aprotinin can inhibit the proteolytic activity of thrombin. A fluorescence and an enzymatic study.. Eur J Biochem.

[20] Bode W, Mayr I, Baumann U, Huber R, Stone SR (1989). The refined 1.9 Å crystal structure of human alpha-thrombin: interaction with D-Phe-Pro-Arg chloromethylketone and significance of the Tyr-Pro-Pro-Trp insertion segment.. EMBO J.

[21] Le Bonniec BF, Guinto ER, MacGillivray RT, Stone SR, Esmon CT (1993). The role of thrombin's Tyr-Pro-Pro-Trp motif in the interaction with fibrinogen, thrombomodulin, protein C, antithrombin III, and the Kunitz inhibitors.. J Biol Chem.

[22] Guinto ER, Ye J, Le Bonniec BF, Esmon CT (1994). Glu192–>Gln substitution in thrombin yields an enzyme that is effectively inhibited by bovine pancreatic trypsin inhibitor and tissue factor pathway inhibitor.. J Biol Chem.

[23] van de Locht A, Bode W, Huber R, Le Bonniec BF, Stone SR (1997). The thrombin E192Q-BPTI complex reveals gross structural rearrangements: implications for the interaction with antithrombin and thrombomodulin.. EMBO J.

[24] Fuentes-Prior P, Iwanaga Y, Huber R, Pagila R, Rumennik G (2000). Structural basis for the anticoagulant activity of the thrombin-thrombomodulin complex.. Nature.

[25] van de Locht A, Stubbs MT, Bode W, Friedrich T, Bollschweiler C (1996). The ornithodorin-thrombin crystal structure, a key to the TAP enigma?. EMBO J.

[26] Wei A, Alexander RS, Duke J, Ross H, Rosenfeld SA (1998). Unexpected binding mode of tick anticoagulant peptide complexed to bovine factor Xa.. J Mol Biol.

[27] Sonenshine DE (1991). The Biology of Ticks, Vol. 1..

[28] Willadsen P, Riding GA (1980). On the biological role of a proteolytic-enzyme inhibitor from the ectoparasitic tick *Boophilus microplus*.. Biochem J.

[29] Willadsen P, McKenna RV (1983). Trypsin-chymotrypsin inhibitors from the tick, *Boophilus microplus*.. Aust J Exp Biol Med Sci.

[30] Viljoen GJ, Neitz AWH, Prozesky L, Bezuidenhout JD, Vermeulen NMJ (1985). Purification and properties of tick egg toxic proteins.. Insect Biochem.

[31] Vermeulen NM, Viljoen GJ, Bezuidenhout JD, Visser L, Neitz AW (1988). Kinetic properties of toxic protease inhibitors isolated from tick eggs.. Int J Biochem.

[32] Tanaka AS, Andreotti R, Gomes A, Torquato RJ, Sampaio MU (1999). A double headed serine proteinase inhibitor--human plasma kallikrein and elastase inhibitor--from *Boophilus microplus* larvae.. Immunopharmacology.

[33] Sasaki SD, Azzolini SS, Hirata IY, Andreotti R, Tanaka AS (2004). *Boophilus microplus* tick larvae, a rich source of Kunitz type serine proteinase inhibitors.. Biochimie.

[34] Fogaça AC, Almeida IC, Eberlin MN, Tanaka AS, Bulet P (2006). Ixodidin, a novel antimicrobial peptide from the hemocytes of the cattle tick *Boophilus microplus* with inhibitory activity against serine proteinases.. Peptides.

[35] Santos IK, Valenzuela JG, Ribeiro JM, de Castro M, Costa JN (2004). Gene discovery in *Boophilus microplus*, the cattle tick: the transcriptomes of ovaries, salivary glands, and hemocytes.. Ann N Y Acad Sci.

[36] Guerrero FD, Miller RJ, Rousseau ME, Sunkara S, Quackenbush J (2005). BmiGI: a database of cDNAs expressed in *Boophilus microplus*, the tropical/southern cattle tick.. Insect Biochem Mol Biol.

[37] Lai R, Takeuchi H, Jonczy J, Rees HH, Turner PC (2004). A thrombin inhibitor from the ixodid tick, *Amblyomma hebraeum*.. Gene.

[38] Antonini E, Ascenzi P, Menegatti E, Guarneri M (1983). Multiple intermediates in the reaction of bovine beta-trypsin with bovine pancreatic trypsin inhibitor (Kunitz).. Biopolymers.

[39] Hynes TR, Randal M, Kennedy LA, Eigenbrot C, Kossiakoff AA (1990). X-ray crystal structure of the protease inhibitor domain of Alzheimer's amyloid beta-protein precursor.. Biochemistry.

[40] Willadsen P, Riding GA (1979). Characterization of a proteolytic-enzyme inhibitor with allergenic activity. Multiple functions of a parasite-derived protein.. Biochem J.

[41] Valenzuela JG, Francischetti IM, Pham VM, Garfield MK, Mather TN (2002). Exploring the sialome of the tick *Ixodes scapularis*.. J Exp Biol.

[42] Francischetti IMB, My Pham V, Mans BJ, Andersen JF, Mather TN (2005). The transcriptome of the salivary glands of the female western black-legged tick *Ixodes pacificus* (Acari: Ixodidae).. Insect Biochem Mol Biol.

[43] Francischetti IMB, Valenzuela JG, Andersen JF, Mather TN, Ribeiro JMC (2002). Ixolaris, a novel recombinant tissue factor pathway inhibitor (TFPI) from the salivary gland of the tick, *Ixodes scapularis*: identification of factor X and factor Xa as scaffolds for the inhibition of factor VIIa/tissue factor complex.. Blood.

[44] Monteiro RQ, Rezaie AR, Ribeiro JMC, Francischetti IMB (2005). Ixolaris: a factor Xa heparin-binding exosite inhibitor.. Biochem J.

[45] Xu Y, Carr PD, Guss JM, Ollis DL (1998). The crystal structure of bikunin from the inter-alpha-inhibitor complex: a serine protease inhibitor with two Kunitz domains.. J Mol Biol.

[46] Nienaber J, Gaspar AR, Neitz AW (1999). Savignin, a potent thrombin inhibitor isolated from the salivary glands of the tick *Ornithodoros savignyi* (Acari: Argasidae).. Exp Parasitol.

[47] Mans BJ, Louw AI, Neitz AW (2002). Amino acid sequence and structure modeling of savignin, a thrombin inhibitor from the tick, *Ornithodoros savignyi*.. Insect Biochem Mol Biol.

[48] Bah A, Chen Z, Bush-Pelc LA, Mathews FS, Di Cera E (2007). Crystal structures of murine thrombin in complex with extracellular fragments of murine protease-activated receptors PAR3 and PAR4.. Proc Natl Acad Sci U S A.

[49] Horn F, dos Santos PC, Termignoni C (2000). *Boophilus microplus* anticoagulant protein: an antithrombin inhibitor isolated from the cattle tick saliva.. Arch Biochem Biophys.

[50] Ciprandi A, de Oliveira SK, Masuda A, Horn F, Termignoni C (2006). *Boophilus microplus*: its saliva contains microphilin, a small thrombin inhibitor.. Exp Parasitol.

[51] Jittapalapong S, Jansawan W, Barriga OO, Stich RW (2004). Reduced incidence of *Babesia bigemina* infection in cattle immunized against the cattle tick, *Boophilus microplus*.. Ann N Y Acad Sci.

[52] Czapinska H, Otlewski J (1999). Structural and energetic determinants of the S1-site specificity in serine proteases.. Eur J Biochem.

[53] Grzesiak A, Krokoszynska I, Krowarsch D, Buczek O, Dadlez M (2000). Inhibition of six serine proteinases of the human coagulation system by mutants of bovine pancreatic trypsin inhibitor.. J Biol Chem.

[54] Grzesiak A, Helland R, Smalas AO, Krowarsch D, Dadlez M (2000). Substitutions at the P(1) position in BPTI strongly affect the association energy with serine proteinases.. J Mol Biol.

[55] Sichler K, Hopfner KP, Kopetzki E, Huber R, Bode W (2002). The influence of residue 190 in the S1 site of trypsin-like serine proteases on substrate selectivity is universally conserved.. FEBS Lett.

[56] Pereira PJB, Bergner A, Macedo-Ribeiro S, Huber R, Matschiner G (1998). Human beta-tryptase is a ring-like tetramer with active sites facing a central pore.. Nature.

[57] Martin PD, Malkowski MG, Box J, Esmon CT, Edwards BF (1997). New insights into the regulation of the blood clotting cascade derived from the X-ray crystal structure of bovine meizothrombin des F1 in complex with PPACK.. Structure.

[58] Adler M, Davey DD, Phillips GB, Kim SH, Jancarik J (2000). Preparation, characterization, and the crystal structure of the inhibitor ZK-807834 (CI-1031) complexed with factor Xa.. Biochemistry.

[59] Francischetti IM, Mather TN, Ribeiro JM (2004). Penthalaris, a novel recombinant five-Kunitz tissue factor pathway inhibitor (TFPI) from the salivary gland of the tick vector of Lyme disease, *Ixodes scapularis*.. Thromb Haemost.

[60] Girard TJ, Warren LA, Novotny WF, Likert KM, Brown SG (1989). Functional significance of the Kunitz-type inhibitory domains of lipoprotein-associated coagulation inhibitor.. Nature.

[61] Venclovas C, Zemla A, Fidelis K, Moult J (2003). Assessment of progress over the CASP experiments.. Proteins.

[62] Brandstetter H, Turk D, Hoeffken HW, Grosse D, Sturzebecher J (1992). Refined 2.3 Å X-ray crystal structure of bovine thrombin complexes formed with the benzamidine and arginine-based thrombin inhibitors NAPAP, 4-TAPAP and MQPA. A starting point for improving antithrombotics.. J Mol Biol.

[63] Maskos K, Huber-Wunderlich M, Glockshuber R (2003). DsbA and DsbC-catalyzed oxidative folding of proteins with complex disulfide bridge patterns *in vitro* and *in vivo*.. J Mol Biol.

[64] Leslie AGW (1992). Recent changes to the MOSFLM package for processing film and image plate data.. Joint CCP4+ESF-EAMCB Newsletter on Protein Crystallography no. 26.

[65] Collaborative Computational Project No. 4 (1994). The CCP4 suite: programs for protein crystallography.. Acta Crystallogr.

[66] Navaza J (1994). AMoRe: an automated package for molecular replacement.. Acta Crystallogr.

[67] Malkowski MG, Martin PD, Guzik JC, Edwards BF (1997). The co-crystal structure of unliganded bovine alpha-thrombin and prethrombin-2: movement of the Tyr-Pro-Pro-Trp segment and active site residues upon ligand binding.. Protein Sci.

[68] Parkin S, Rupp B, Hope H (1996). Structure of bovine pancreatic trypsin inhibitor at 125 K definition of carboxyl-terminal residues Gly57 and Ala58.. Acta Crystallogr.

[69] Brünger AT, Adams PD, Clore GM, DeLano WL, Gros P (1998). Crystallography & NMR system: A new software suite for macromolecular structure determination.. Acta Crystallogr.

[70] Winn MD, Isupov MN, Murshudov GN (2001). Use of TLS parameters to model anisotropic displacements in macromolecular refinement.. Acta Crystallogr.

[71] Barton GJ (1993). ALSCRIPT: a tool to format multiple sequence alignments.. Protein Eng.

[72] Schwede T, Kopp J, Guex N, Peitsch MC (2003). SWISS-MODEL: An automated protein homology-modeling server.. Nucleic Acids Res.

[73] Evans SV (1990). SETOR: Hardware lighted three-dimensional solid model representations of macromolecules.. J Mol Graphics.

[74] Siekmann J, Wenzel HR, Schroder W, Schutt H, Truscheit E (1987). Pyroglutamyl-aprotinin, a new aprotinin homologue from bovine lungs--isolation, properties, sequence analysis and characterization using 1H nuclear magnetic resonance in solution.. Biol Chem.

[75] Mine S, Yamazaki T, Miyata T, Hara S, Kato H (2002). Structural mechanism for heparin-binding of the third Kunitz domain of human tissue factor pathway inhibitor.. Biochemistry.

[76] Merigeau K, Arnoux B, Perahia D, Norris K, Norris F (1998). 1.2 Å refinement of the Kunitz-type domain from the alpha3 chain of human type VI collagen.. Acta Crystallogr.

