# Supplementary material for: Isolation, Cloning and Structural Characterisation of Boophilin, a Multifunctional Kunitz-Type Proteinase Inhibitor from the Cattle Tick
Source: PLoS One. 2008 Feb 20;3(2):e1624. doi: 10.1371/journal.pone.0001624 (PMC2230226; doi:10.1371/journal.pone.0001624)
Supplement: Supplementary file 1 [file pone.0001624.s003.doc]

**Peptide**	**Sequence**
CNBr	FSYGGCGGNENNFETIEDCQKACGAP
Lys-C5	ACGAPERVNDFESADFK
Lys-C6	ACGAPERVSDFEGADFK
Lys-C11	ALIPRFYFNTETGK
Lys-C15	TGCEPAADSGSCAGQLERWFYNVRSGECETFVYGGCS
Asp-N1	DFEGA
Asp-N2	DFKTGCEPA
Asp-N3	DFKTGC
Asp-N4	DCQKACGAPER
Asp-N5	DSGSCAGQ
Asp-N6	ELVCKN
Asp-N7	ETFVYGGCGGN
Asp-N8	DNNYES
