# Supplementary material for: Isolation, Cloning and Structural Characterisation of Boophilin, a Multifunctional Kunitz-Type Proteinase Inhibitor from the Cattle Tick
Source: PLoS One. 2008 Feb 20;3(2):e1624. doi: 10.1371/journal.pone.0001624 (PMC2230226; doi:10.1371/journal.pone.0001624)
Supplement: Supplementary file 2 [file pone.0001624.s004.doc]

**Primer**	**Sequence**
Lys-C11Fwd	5’-GCGGATCCTTYTAYTTYAAYACNGARACNGG-3’
Lys-C15Rev	5’-GCGGATCCCANCCNCCRTANACRAANGTYTC-3’
OmpA-Fwd	5’-ATTTCTAGATAACGAGGGCAAAAA-3’
OmpA-Rev	5’-TCGGCAGAATCCATTTCTCTGGGCCTGCGCTACGGTAGCGAA-3’
Booph-Fwd	5’-TTCGCTACCGTAGCGCAGGCC**CAG**AGAAATGGATTCTGCCGA-3’
Booph-Rev	5’-TTTTCAGAAGCTTA**TTA**CATGTTCTTGCAGACGAG-3’
